# The study of targeted blocking SDF-1/CXCR4 signaling pathway with three antagonists on MMPs, type II collagen, and aggrecan levels in articular cartilage of guinea pigs

**DOI:** 10.1186/s13018-020-01646-1

**Published:** 2020-05-29

**Authors:** Guoliang Wang, Yanlin Li, Xuhan Meng, Xiao Yang, Yaoyu Xiang

**Affiliations:** 1 Department of Sports Medicine, First Affiliated Hospital of Kunming Medical University, No.295 Xichang Road, Kunming, 650031 Yunnan China; 2 Kunming Medical University, No.1168 Chunrong Road, Chenggong District, Kunming, 650500 Yunnan China

**Keywords:** Osteoarthritis, Chondrocyte, Stromal cell derived factor-1(SDF-1), Matrix metalloproteinase

## Abstract

**Objective:**

To explore the possibility and mechanism of targeted blocking SDF-1/CXCR4 signaling pathway using three antagonists TN14003, T140, and AMD3100 in vivo, and to investigate the function of three antagonists in delay degeneration process of articular cartilage.

**Methods:**

Ninety-six male Duncan-Hartley guinea pigs (6 months old) were divided into groups A, B, C, and D randomly. Alzet trace pump was implanted in the back subcutaneous tissue of pigs in group A, and TN14003 with concentration of 180 μg/ml was pumped every day. Alzet trace pump was implanted in the back subcutaneous tissue of pigs in group B, and T140 with concentration of 180 μg/ml was pumped every day. Alzet trace pump was implanted in the back subcutaneous tissue of pigs in group C, and AMD3100 with concentration of 180 μg/ml was pumped every day. Hartley guinea pigs in group D remained untreated as the blank control group. At 2, 4, 6, 8, 10, and 12 weeks of treatment, 5 to 8 animals in each group were randomly chosen for blood collection via cardiac puncture. SDF-1 content using enzyme-linked immunosorbent assay (ELISA). At 12 weeks, all guinea pigs were sacrificed by injecting pentobarbital sodium (30 mg/kg) into the peritoneal cavity. Cartilages from the tibial plateau in each group were harvested for PCR testing and western blot analysis. SPSS19.0 was used for data analysis.

**Results:**

Result of ELISA: the serum levels of SDF-1 of groups A, B, and C decreased gradually with time. Significant drop of SDF-1 level was seen in group A while increased SDF-1 was shown in group D. At the same time, the serum levels of SDF-1 of the group A were significantly lower than that of group B; those of group B were significantly lower than that of group C, which was significantly lower than that of group D, and their difference is statistically significant (*P* < 0.05). Real time quantitative PCR result: The mRNA levels of MMPs in group A were significantly lower than group B, and those of group B were significantly lower than group C, which was significantly lower than group D, and there was statistically significant (*P* < 0.05). The mRNA levels of type II collagen, aggrecan in group A were significantly more than group B; those of group B were significantly more than group C, which was significantly more than group D, and the difference was statistically significant (*P* < 0.05). H&E staining result: cartilage of group C was more significantly degenerative than other groups.

**Conclusions:**

The three antagonists can target SDF-1/CXCR4 signaling pathway in vivo, reduce the expression and secretion of MMP-3, MMP-9, and MMP-13 in cartilage tissue, and reduce the degradation of collagen II and aggregating proteoglycan, thus delaying the degeneration of articular cartilage, of which TN14003 has the strongest regulatory effect. Targeted blockade of SDF-1/CXCR4 signaling pathway by TN14003 in vivo delays articular cartilage degeneration more effectively than T140 and AMD3100.

## Introduction

Osteoarthritis(OA) is a common chronic osteoarthropathy with joint cartilage damage and involves subchondral bone and synovial membrane degeneration, accompanied by osteoporosis and osteophyte [1–4]. Under normal circumstances, chondrocyte can secrete a large number of substances, including synthetic collagen II, aggrecan, and other substances secreted to the extracellular cell matrix (ECM), constituting an extracellular fiber network component, providing support and protection, lubrication, and nutrition for cells [5]. At the same time, when cartilage is damaged, the amount of MMPs released by cartilage tissue increases, which leads to the decrease and degradation of ECM, the destruction of extracellular fibrous network of cartilage tissue, and the further aggravation of cartilage tissue damage, leading to the occurrence of OA. SDF-1 plays a critical role in the pathological cartilage degeneration process of patients with OA. Blocking agent (AMD3100, T140) can stop SDF-1 in combination with CXCR4 on the cartilage surface, which can activate the signal pathway of extracellular signal regulator enzyme (Erk) and related kinase (p38 MAP kinase), thus inhibiting the secretion of MMP-3, MMP-9, and MMP-13 by cartilage tissue, reducing the degradation of type II collagen and aggregating proteoglycan, leading to the reduction of articular cartilage damage [6, 7]. However, AMD3100 has toxic side effects, and T140 serum is unstable, while TN14003, as a derivative of T140, has better serum stability and lower cytotoxicity, thus its clinical application prospect is broad [8, 9]. TN14003 provides a theoretical basis for further exploration of CXCR4 target antagonist with high efficiency and small side effects, and provides a basis for the search for targeted drugs for OA.

In this study, 6-month-old male Hartley guinea pigs were injected with three types of CXCR4 specific antagonists. Alzet trace pump lasted up for 12 weeks to block SDF-1/CXCR4 signaling pathway. ELISA was used to determine the levels of SDF-1 in serum; real time quantitative PCR was used to test the expression of MMP-3,9,13; aggrecan and type II collagen mRNA, and western blot to test type II collagen in the cartilage tissue.

## Materials and methods

### Animals and groups

Duncan-Hartley guinea pigs develop spontaneous knee OA at around age of 6 months [4]. Ninety-six male Duncan-Hartley guinea pigs (6-month-old, weight = 600 ± 50 g) were obtained from the Institute of Zoology, Chinese Academy of Sciences (Kunming, China). All guinea pigs were fed in tray type cages with sawdust as bedding material; the sawdust was renewed every day to keep the living environment dry. Every four guinea-pig were housed and fed in one cage. Clean water in a lick type bottle was supplied. The breeding room was kept quiet to avoid frightening animals. A light and dark cycle was formed by turning on the sunlight lamp at 08.00 h and off at 20.00 h. All animal experiments were performed in accordance with the approval of the Institutional Committee on the Care and Use of Animals of Kunming Medical University (issue 2017/03).

Ninety-six male Duncan-Hartley guinea pigs (6 months old) were divided into groups A, B, C, and D randomly. Alzet trace pump was implanted in the back subcutaneous tissue of the pigs in group A, and TN14003 with concentration of 180 μg/ml was pumped every day. Alzet trace pump was implanted in the back subcutaneous tissue of pigs in group B, and T140 with concentration of 180ug/ml was pumped every day. Alzet trace pump was implanted in the back subcutaneous tissue of pigs in group C, and AMD3100 with concentration of 180 μg/ml was pumped every day. Hartley guinea pigs in group D received no treatment. In the 2nd, 4th, 6th, 8th, 10th, and 12th week, the levels of SDF-1 in serum were quantified with ELISA. All the pigs were sacrificed after 12-week conventional breeding, and the cartilage of both knees of each pig was removed immediately. Real time quantitative PCR was used to test the expression of MMP-3, MMP-9, and MMP-13; aggrecan; and type II collagen mRNA, and western blot to test type II collagen in the cartilage tissue, and SPSS19.0 was used for data analysis.

### Mini-osmotic pump implantation and surgical procedures

Guinea pigs were anesthetized with ketamine (50 mg/kg body weight) and xylazine (5 mg/kg body weight). An area (~ 4 cm^2^) of the back over the dorsolateral thorax in group A and B animals was clipped and prepared for aseptic surgery. After disinfection using 75% alcohol, the skin was cut a small incision (~ 1 cm), and a small subcutaneous pocket was formed by blunt dissection. A mini-osmotic pump was embedded into this pocket. Before implanting the pumps to Guinea pigs, each pump was filled into 200 μl of solution containing 50 mg/ml TN1400 in PBS (group A) or 50 mg/ml T140 in group B and 50 mg/ml AMD3100 in PBS (group C) and PBS alone (group D). At an average pumping rate of 0.15 μl/h, each animal in groups A, B, and C received 180 μg of TN14003, T140, and AMD3100 per day, respectively. The pumps were exchanged once after 6 weeks from the date of implantation.

### Sample collection

At 2, 4, 6, 8, 10, and 12 weeks of treatment, 5 to 8 pigs in each group were randomly chosen for blood collection using cardiac puncture. Blood samples were centrifuged for 5~10 min (3000 r/min) to remove cells and debris within an hour after sample collection, and supernatants were sterilized using 0.22 μm millipore filters and injected into centrifugal tube, and stored in a − 80 °C cryogenic refrigerator for later detection of SDF-1 content using enzyme-linked immunosorbent assay (ELISA). At 12 weeks, all guinea pigs were sacrificed by injecting pentobarbital sodium (30 mg/kg) into the peritoneal cavity. Cartilages from the tibial plateau in each group were harvested for histological assay, PCR testing.

### ELISA

Serum SDF-1 in each group was analyzed using an ELISA kit (R&D Systems, Minneapolis, USA) according to the manufacturer’s protocol. ELISA assays were performed in duplicate wells for each sample. Optical density values in developed plates were determined using a microplate reader set to 450 nm; then, depending on linear regression equation standards, we calculated the concentration of SDF-1 in samples.

### Reverse transcription-polymerase chain reaction assay

Total RNA was isolated from tibial plateau cartilage tissue with a RNeasy isolation kit (Fermentas, Burlington, Canada), and the quantity and purity of extracted RNA was evaluated using a spectrophotometer. One microgram of total RNA was transcribed into cDNA according to RevertAid^TM^ First Strand cDNA Synthesis Kit instructions (Fermentas, Burlington, Canada), and 40 ng/μl of this obtained cDNA was used as a template for PCR amplification in order to quantify the relative content of each mRNA using a LightCycler480 SYBR Green I Master (Roche Diagnostics, Mannheim, Germany). β-Actin was used as an internal reference. Primers for β-Actin, MMP-3, MMP-9, MMP-13, ACAN, and Col II designed by OMIGA v2.0 (Genetics Computer Group, Madison, USA), were used to amplify products contained within the coding sequence of the various RNAs. Expression levels were measured in triplicate. Primer sequences are listed in Table 1. The data are given as a threshold cycle (Ct). Fold changes in gene expression were calculated to be 2^−Δ(ΔCt)^. The comparative Ct (threshold cycles) method was used to evaluate the expression level of each target gene relative to the level in the untreated group.

**Table 1:** Sequences of primers used for RT-PCR

Gene	Sequences of primers	Tm(°C)	Length (bp)
ACTIN	F: 5'-CCACCATGTACCCAGGCATT-3'	60 °C	177 bp
	R: 5'-ACTCCTGCTTGCTGATCCAC-3'		
CollI	F: 5'-ATGCACCTTGGATGCCATGA-3'	59 °C	103 bp
	R: 5'-GCTGCTCCACCAGTTCTTCT-3'		
MMP3	F: 5'-GGACAAAGGATACAACAGGGAC-3'	57 °C	157 bp
	R: 5'-TCATCTTGAGACAGGCGAAA-3'		
MMP9	F: 5'-AGGAAAGGCGCTGCTCTTCA-3'	60 °C	103 bp
	R: 5'-GGAGAACACATGGTCCACCG-3'		
MMP13	F: 5'-CGCTACCTGAAATCATACTACCA-3'	57 °C	122 bp
	R: 5'-CCTGTCACCTCTAAGCCAAAG-3'		
Acan	F: 5'-ACATCTCAGCAGCATCATCACC-3'	59 °C	199 bp
	R: 5'-CATCACCACGCAGTCCTCAC-3'		

### Western blot

Western blot 100 mg of frozen cartilage tissue was dissected and immediately transferred to the RIPA buffer with a protease inhibitor cocktail, and then homogenized on ice using the SilentCrusher M Homogenizer. The homogenate was centrifuged (12,000 rpm for 10 min at 4 °C). The supernatant was collected and quantified using a BAC Protein Assay Kit (Pierce, Rockford, USA). In brief, 10 μg of total protein was electrophoresed in 10% SDS-PAGE, and proteins from sodium dodecyl sulfate (SDS) gels were electrophoretically transferred to a polyvinylidene difluoride membrane (Millipore, Bedford, USA) and blocked with 5% nonfat milk in tris-buffered saline-Tween 20 (TBST). Membranes were probed by a human anti-Col II monoclonal antibody (1:1000 dilution; Proteintech Group, Chicago, USA) and anti-β-actin polyclonal antibody (1:1000 dilution; Cell Signaling Technology, Danvers, USA). Horseradish peroxidase-conjugated goat anti-rabbit immunoglobulin G (IgG) (H + L) (1:3000 dilution, Bio-Rad Laboratories, Richmond, USA) was used as the secondary antibody. Chemiluminescence was visualized by a Tanon-4500 digital image processing system (Tanon, Shanghai, China), and light was then detected by photographic film.

### Statistical analysis

Statistical analysis was performed using SPSS v19.0 (SPSS Inc, Chicago, USA). All data are presented as mean ± standard deviation (SD). All data conform to the normal distribution. ANOVA for repeated measurement data and Student’s *t* test were used for statistical analysis. *P* values < 0.05 were considered statistically significant.

## Results

### Three antagonists decreased SDF-1 in serum

**Fig 1:**
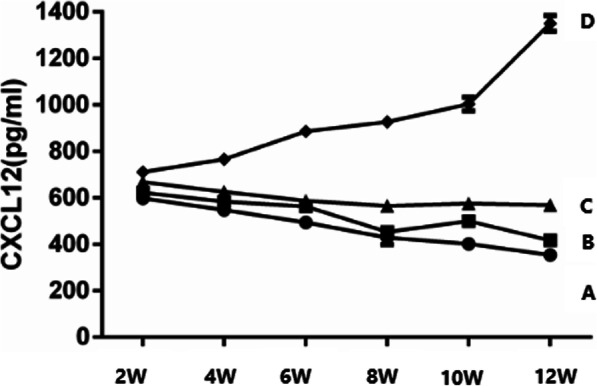
Levels of SDF-1 in the serum of guinea pigs in four groups measured using ELISA

The level of SDF-1 in serum of groups A, B, and C decreased gradually, with the most significant decrease in group A and gradual increase in group D. At the same time point, the level of SDF-1 in serum of group A was lower than that of group B, and SDF-1 level of group B was lower than that of group C, and for group C, the value was lower than that of group D; the difference was statistically significant (*P* < 0.05) (Table 2). With the prolonged intervention time of the three antagonists, the level of SDF-1 in the serum of groups A, B, and C gradually decreased, with the most significant decrease in group A and gradual increase in group D (Fig. 1).

**Table 2:** Comparison of SDF-1 ELISA in serum($\overline{X}$ ± S, *n* = 24)($\overline{X}$ ± SD, *n* = 24)pg/mL

Group	2week	4week	6week	8week	10week	12week
A	597.35±3.82	547.35±5.55	493.62±13.89	428.48±29.59	401.59±20.17	354.04±7.59
B	622.50±9.52	583.78±8.20	562.66±4.79	453.59±13.15	500.29±18.04	416.68±14.98
C	667.91±1.82	625.47±10.12	587.35±11.48	565.71±3.99	575.98±11.72	568.56±6.80
D	711.24±8.08	766.03±7.12	886.43±21.98	927.07±11.68	1003.50±29.44	1350.44±33.57
F	644.47	643.78	618.74	583.13	616.17	742.51
P	0.000	0.000	0.000	0.000	0.000	0.000

Comparing with group A at each time point, *P* < 0.05;Compared with group B, *P* < 0.05; Compared with group C, *P* < 0.05; Compared with group D, *P* < 0.05

As time went on, the level of SDF-1 in serum of groups A, B, and C decreased gradually, with the most significant decrease in group A and a gradual increase in group D. At each measured time point, the sequence of SDF-1 contents in serum were successively A < B < C < D.

### Three antagonists downregulated the expression of MMP-3, MMP-9, and MMP-13 and upregulated the expression of Col II and aggrecan

**Fig. 2:**
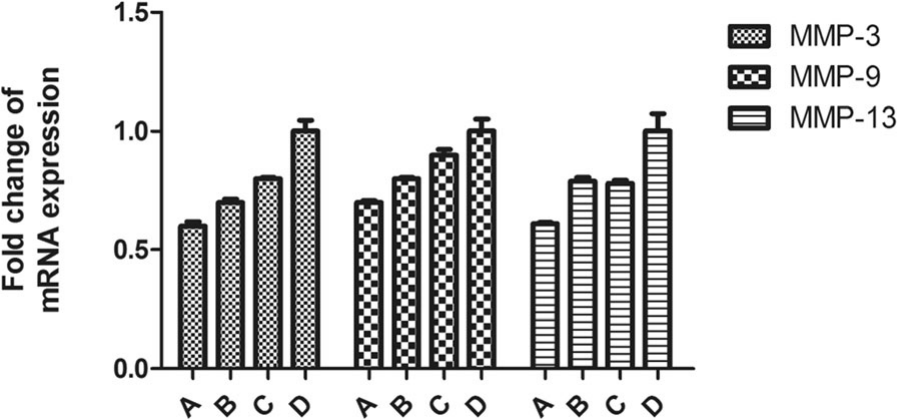
The expression of MMP-3, MMP-9, and MMP-13 measured using RT-PCR

**Fig. 3:**
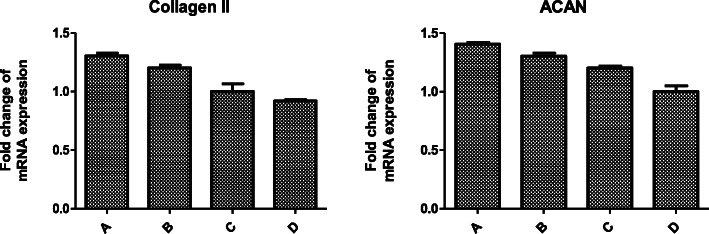
The expression of Col II and aggrecan measured using RT-PCR

To investigate the effect of three antagonists on cartilage degeneration, we examined the expression patterns of the cartilage-degrading proteases MMP-3, MMP-9, and MMP-13, and the cartilage matrix proteins Col II and aggrecan in cartilage. We found that mRNA expression levels of mmp-3, mmp-9, and mmp-13 in group A were lower than those in group B (*P* < 0.05), and the level in group B was lower than that in group C (*P* < 0.05), group C was lower than that in group D (*P* < 0.05), and the difference was statistically significant (*P* < 0.05). The mRNA expression patterns of these five genes in each group compared with those in the other groups were statistically different (*P* < 0.05), and mRNA expression of Col II and ACAN showed the opposite result (Figs. 2 and 3).

Figures 2 and 3 show the expression of MMP-3, MMP-9, MMP-13, Col II, and aggrecan measured using RT-PCR. Fold changes in gene expression were calculated as 2^−Δ(ΔCt)^. Histogram analysis shows that the mRNA expression levels of mmp-3, mmp-9, and mmp-13 in group A were lower than those in group B (*P* < 0.05), and the level in group B was lower than that in group C (*P* < 0.05), group C was lower than that in group D (*P* < 0.05), and the mRNA expression patterns of these five genes in each group compared with those in the other groups were statistically different (*P* < 0.05).

### Three antagonists reduced degradation of type II collagen in the cartilage matrix

**Fig. 4:**
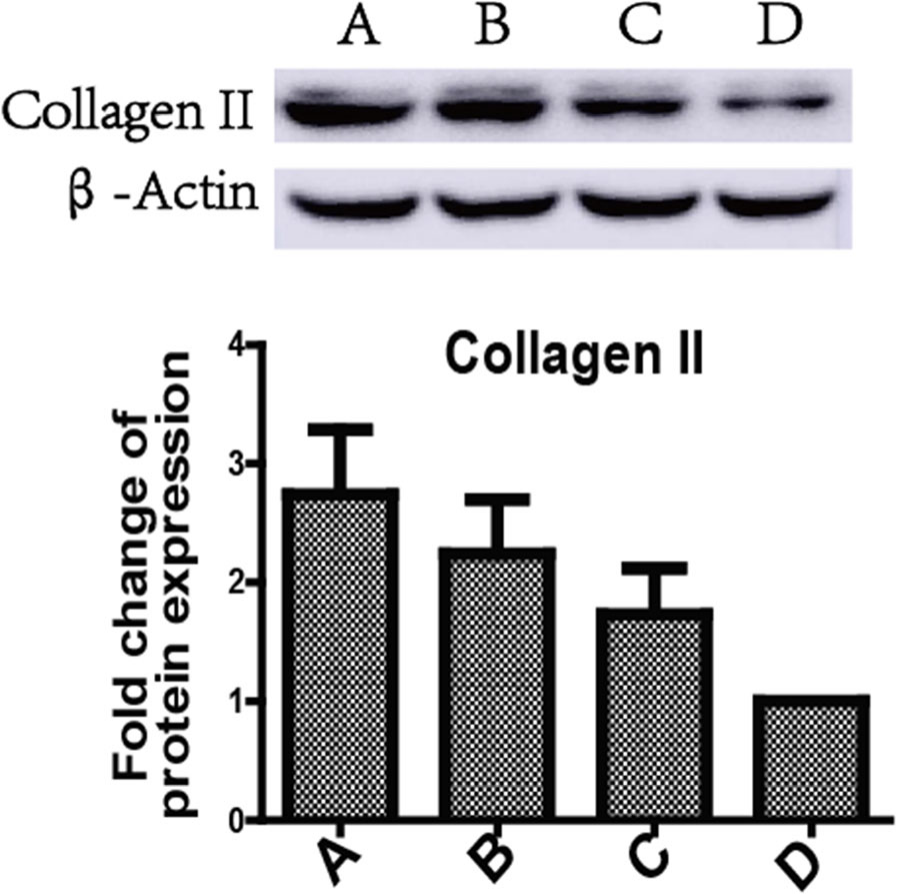
Three antagonists reduced degradation of type II collagen in the cartilage matrix

**Fig. 5:**
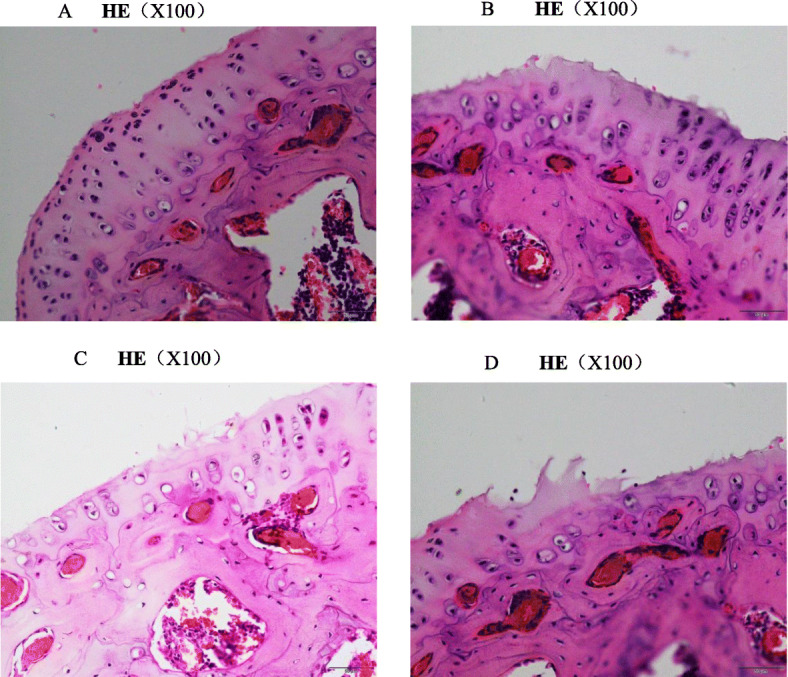
The H&E staining. In groups A, B, and C, we found that the chondrocytes were closely arranged, and nuclei were stained homogeneously, in addition, there were more cells. But in group D, there were exfoliation of exterior cells, and chondrocytes were irregular and arranged disorderly; nuclei were stained unhomogeneously and less quantity of cells

Three antagonists reduced degradation of type II collagen in the cartilage matrix. Given that type II collagen was the main component of the cartilage matrix, we determined the effects of three antagonists on type II collagen. We found that the expression in group A was the most followed by group B, and group C was higher than group D. Group A had significant difference compared with other groups (*P* < 0.05). Col II protein levels in each group compared with those in the other groups were statistically different (*P* < 0.05) (Figs. 4 and 5).

## Discussion

The SDF-1 (also known as chemotactic factor) is a CXC small molecule chemotactic protein produced by a variety of cells. It belongs to the family of chemokines proteins, whose receptors are CXCR4, and are able to form specific bindings [10].Through immunohistochemistry test, CXCR4 can be found in both the surface and the deep layer of articular cartilage of an OA patient [11].

Recent studies have shown that SDF-1 is a key cellular factor that leads to osteoarthritis, which may be closely related to the inflammatory response process [4, 12]. MMP-3, MMP-9, and MMp-13 matrix metalloproteinases can be secreted by inflammatory cells and synovial tissue, and these three proteases are the enzymes with strong decomposition function in their enzyme families. They are involved in the degradation of cartilage matrix throughout the pathological mechanism, which can promote the exposure of collagen in cartilage and rapidly destroy the network structure of collagen tissue (“Rigid Structure” of cartilage tissue), and MMP-3 also hydrolyzes collagen that has been split. As the normal cartilage collagen reticular structure loses its “rigidity,” chondrocytes lose their inherent protective and nutritional functions. With the friction and inflammatory mediation of joint activity, it produces cell exfoliation, spindle deformation of chondrocyte, fibrosis, and so on, i.e., irreversible cartilage degeneration [13, 14]. Our previous study found that SDF-1/CXCR4 signaling pathway plays a key role in the occurrence and development of OA, and CXCR4 receptor antagonist has been used in the research of the prevention and treatment of OA. AMD3100 is a small molecule non-peptide inhibitor, whose chemical name is bridging 1,4,8,11-tetra-azacyclopentadecane, and it is a partial blocker of SDF-1/CXCR4 signaling pathway and mainly by binding to the second extracellular ring region of CXCR4 and the residues of its hydrophobic group, specifically blocking CXCR4 binding to its ligand SDF-1 to block SDF-1/CXCR4 signaling pathway [14]. Thus, it can inhibit the signal pathway of extracellular signal regulation enzyme (Erk) and related kinase (p38MAP Kinase); reduce the secretion and release of mmp-3, mmp-9, and mmp-13; and reduce the degradation of cartilage tissue. AMD3100 is a CXCR4 receptor antagonist that is tightly bounded and is slowly reversible. It has a partial role as a weak agonist, and its blocking effect is incomplete. It belongs to CXCR4 incomplete antagonist [13]. T140 (peptide derivative) is a small molecule peptide antagonist of 14 amino acid polypeptides and a complete CXCR4 antagonist [15], which can effectively block SDF-1/CXCR4 signaling pathway. TN14003 is a derivative of T140, it is also a kind of small molecular peptide containing 14 amino acid polypeptides [16]. It has good biological stability and low toxicity after modification such as amination of the hydroxyl terminal of T140. Based on the previous experiment result [16], the target blocker of SDF-1/CXCR4 signal pathway was explored, and experiments in vivo were conducted to further explore the effect of three antagonists to delay or prevent the degeneration of articular cartilage.

Our research found that the level of SDF-1 in serum of groups A, B, and C decreased gradually, with the most significant decrease in group A and gradual increase in group D. At the same time point, the level of SDF-1 in serum of group A is significantly lower than that of group B; group B was lower than that of group C, and group C was lower than that of group D. These results suggest that AMD3100, T140, and TN14003 can reduce the secretion of SDF-1 by blocking the SDF-1/CXCR4 signaling pathway, thus delaying a series of pathological processes leading to OA. With the prolonged intervention time of the three antagonists, the level of SDF-1 in the serum of groups A, B, and C decreased gradually, with the most significant decrease in group A and gradual increase in group D, indicating that three antagonists played an important role in delaying the degeneration of articular cartilage, and group A had the greatest influence. The mRNA expression levels of mmp-3, mmp-9, and mmp-13 in group A were lower than those in group B; group B was lower than that in group C; group C was lower than that in group D, blocking SDF/CXCR4 signaling pathway, which can reduce the secretion and release of MMP-3, MMP-9, and MMP-13 by chondrocytes, and reduce the destruction of type II collagen and aggrecan, thereby delaying the degeneration of articular cartilage. Especially TN14003 had the strongest effect in reducing cartilage degeneration.

To sum up, three antagonists AMD3100, T140, and TN14003 block SDF-1/CXCR4 signaling pathway to reduce the secretion of matrix metalloproteinase, thereby reducing the degeneration of articular cartilage.TN14003 has stronger effect than T140 and AMD3100. The SDF-1/CXCR4 signaling pathway can be a target to prevent the degeneration of articular cartilage. AMD3100 has toxic side effects and T140 leads to serum instability; however, TN14003, as a derivative of T140, has good biological stability and low toxicity and has a promising clinical application prospect. It provides a new theoretical basis for further exploring CXCR4 targeting antagonists with high efficiency and low side effects, and provides a basis for OA to find targeted drugs.

## Data Availability

All data generated or analyzed during this study are provided within this article.

## Abbreviations

CXCR4: CX Chemokine receptor 4
OA: Osteoarthritis
ECM: Extracellular matrix
SDF-1: Stromal cells derived factor 1
MMPs: Matrix metalloproteinases
ELISA: Enzyme-linked immunosorbent assay
PCR: Polymerase chain reaction
PBS: Phosphate buffer saline
